# New Techniques in the Study of the Brain Development in Newborn

**DOI:** 10.3389/fnhum.2014.01069

**Published:** 2015-01-20

**Authors:** Matteo Giampietri, Laura Bartalena, Andrea Guzzetta, Antonio Boldrini, Paolo Ghirri

**Affiliations:** ^1^Department of Maternal and Child Health, Division of Neonatology and Neonatal Intensive Care Unit, S. Chiara Hospital, University of Pisa, Pisa, Italy; ^2^Department of Developmental Neuroscience, Stella Maris Scientific Institute, Pisa, Italy

**Keywords:** magnetic resonance imaging, cranial ultrasound, neurodevelopmental outcome, diffusion tensor imaging (DTI), tractography

In the last few decades, the survival rates of preterm babies and full-term babies with severe diseases have increased due to advances in perinatal care. Understandably however, higher survival rates have not been accompanied by an overall reduction of morbidity, so that limitation of long-term neurodevelopmental abnormalities remains a major challenge of early care (Plaisier et al., 2014). The possibility to better predict the outcome of newborns at neurodevelopmental risk is essential to inform early intervention, to allow best allocation of resources, and to minimize long-term consequences. Unfortunately, clinicians continue to possess limited ability to predict neurodevelopmental outcomes, mainly relying, in most settings, on early findings at cranial ultrasound (cUS).

Recent studies (Smyser et al., 2012) have proven the power of magnetic resonance imaging (MRI) superior to other neuroimaging modalities, including cUS, in detecting cerebral injury. Neonatal MRI provides non-invasive, high-resolution images in less than 1 h; scans are performed without sedation eliminating the risk and the costs associated to it and are not associated to radiation exposure, as for computerized tomography (CT). The application of MRI in the neonatal population is rapidly increasing, making MRI one of the key diagnostic tools for the assessment of early brain development and injury.

In specific clinical groups, such as for example very preterm infants, cerebral MRI should become part of standard clinical care and should be systematically performed at term equivalent age (TEA). Accurate assessment of cortical folding at TEA provides an important marker for structural brain growth and maturation. Myelination of the posterior limb of the internal capsule (PLIC) at around 36–38 weeks gestation, identifiable on T1 but also on T2-weighted images, is another important maturational hallmark, since its presence and symmetry are very powerful in predicting motor outcome. MR imaging is superior to cUS also in detecting diffuse white matter (WM) injury. Indeed, although cystic periventricular leukomalacia is seen less often, diffuse non-cystic types of WM injury, including punctate WM lesions and diffuse excessive high signal intensity, are most frequent and are considered the leading cause of disturbed brain growth, connectivity, and functionality. The predictive power of conventional MRI in this domain remains relatively low, as it is not sensitive enough to analyze changes in microstructure; however, it is greatly enhanced by the use of advanced MR techniques targeting the WM, such as diffusion tensor imaging (DTI), that can help analyzing brain growth in extremely preterm babies in the absence of evident WM abnormalities (Ramenghi et al., 2009).

Diffusion tensor imaging (DTI) is a relatively new MR modality that assesses water diffusion in biological tissues at microstructural level. The diffusion tensor describes an ellipsoid in space characterized by the diffusion eigenvalues (λ_1_,λ_2_,λ_3_) in the three orthogonal directions and their corresponding eigenvectors. In brain WM, axial diffusivity (λ_1_) is oriented along the direction of the main tracts and radial diffusivity (λ_2_ and λ_3_) is oriented perpendicular to these tracts. Average diffusivity (*D*_av_) reflects the mean of these eigenvalues and it is an indicator of brain maturation and/or injury. *D*_av_ decreases with increasing age probably for decreasing water content and increasing complexity of WM structures with myelination. Fractional anisotropy (FA) reflects the variance of the eigenvalues, ranging from 0 (isotropic diffusion) to 1 (anisotropic). The diffusion is mainly anisotropic because the water molecules preferentially move in the direction of fascicles of axons (Adams et al., 2010). In the white and gray matter, there is similar water content but different *D*_av_ value probably because the WM is less restrictive to water motion. Brain water content decreases with increasing gestational age and this mostly increases the WM anisotropy values. This increase has also been attributed to changes in WM structure associated with histologic maturation, and it takes place at different rates in different brain areas [the main areas analyzed are in commissural tracts, the corpus callosum (CC), and in projection tracts, the corticospinal tracts (CSTs)]. Developmental changes in anisotropy of cerebral cortex reflect changes in its microstructure, such as the arborization of basal dendrites of cortical neurons, the innervation of the cortical plate by thalamocortical and cortico-cortical fibers, all processes which are important basis of later functional connectivity (Huppi and Dubois, 2006). Because there are strongly preferred directions of diffusion, it is possible to create color maps of neonatal brain with diffusion tensor post-processing techniques. The color maps are based on major orientation with red representing right–left, green representing antero–posterior, and blue representing superior–inferior anatomical directions (De Bruïne et al., 2013) (Figure 1).

**Figure 1:**
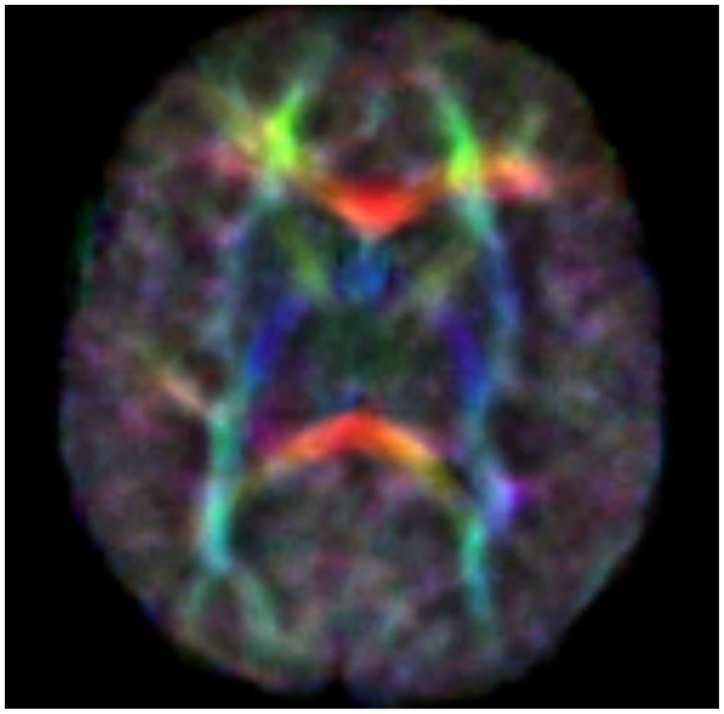
**Color anisotropy maps**.

Preterm birth can cause white matter injuries (WMIs) and consequently can cause change in FA and diffusivity. Decreased FA in the CC of preterm babies scanned at TEA is rather common and implies less efficient transmission between the hemispheres and may lead to language problems and cognitive dysfunctions. Regions with increased FA in a preterm baby may be attributed to a loss or to an impairment of WM instead of improved WM maturation (Li et al., 2014). Disorders of motor function can be tested in clinical practice with DTI. In children with congenital hemiparesis, there are different diffusion characteristics of CSTs compared to healthy one. There is an increasing FA asymmetry and a decrease in FA value in the affected pyramidal tract.

A recent extension of DTI is tractography, which is a powerful tool that offers the possibility of non-invasive identification of specific WM pathways and connections in the brain. The general principle is to connect adjacent image voxels following water diffusion. Directional coherence of the fibers in a pathway is used to determine the presence or absence of connectivity between two regions of the brain. Tracking of the fiber-trajectories is terminated when they turn of too much degrees between two successive voxels. The main regions of interest include the CSTs, the CC, and optic radiations (OR). The primary goal should be to understand the normal relationship between structural and functional networks of these structures but there are few data in preterm babies (Brown et al., 2014). Preterm birth correlates with reduced connectivity, and it is very difficult to establish normal value for all gestational ages. Maturation does not occur simultaneously in the brain infact, for example, connectivity increases earlier in the occipital lobe and then in the frontal area. The postnatal age and WMI are additional confounding factors of diffusion metrics (Pannek et al., 2014). Nevertheless, the primary difference between DTI and conventional imaging is the capability of DTI to often detect injury earlier. This could anticipate the diagnosis of brain damage and might offer advantages in the future for deciding early intervention or administration of neuroprotective agents. Further studies will be needed to confirm whether these new techniques may predict neurodevelopmental outcome and whether they are equally applicable to all the pathways of the central nervous system.

## Conflict of Interest Statement

The authors declare that the research was conducted in the absence of any commercial or financial relationships that could be construed as a potential conflict of interest.
